# Thinking inside the box: do hiding boxes reduce the fear and stress of hospitalised cats?

**DOI:** 10.18849/ve.v10i4.727

**Published:** 2025-12-01

**Authors:** Leah Foster+

**Keywords:** BEHAVIOUR, FELINE, HIDING BOXES, SENSORY ENRICHMENT, STRESS RELIEF, WELLBEING

## Abstract

**PICO question:**

In hospitalised domestic cats, does the use of hiding boxes compared to no intervention reduce fear and stress?

**Clinical bottom line:**

**Category of research:**

Treatment.

**Number and type of study designs reviewed:**

Five studies were critically reviewed. All five studies were defined by the researchers as randomised control trials.

**Strength of evidence:**

Moderate.

**Outcomes reported:**

Four of the five studies showed moderate evidence that hiding boxes reduce fear and stress in cats, with reduced cat stress scores (CSS) in cats given a hiding box compared to control groups. The hiding box was found more useful in aggressive cats as CSS reduced faster in these groups. Among the three studies that recorded objective physiological stress measures (e.g., temperature, salivary cortisol), two studies showed no statistically significant stress reductions between the hiding box and control groups. Only one study of these three found lower heart rates in hiding box cats, but this may have been influenced by confounding factors.

**Conclusion:**

There is moderate evidence suggesting that the use of hiding boxes in hospitalised cats is associated with reduced stress. The strength of the evidence is considered moderate due to varying limitations (e.g., small sample sizes) in the reviewed studies. Despite these limitations, incorporating a plastic or single-use disposable cardboard hiding box into existing feline-friendly cage environments is recommended for consideration. However, the use of a hiding box should be determined on a case-by-case basis as it may not be suitable for all patients (e.g. critical care patients requiring constant visualisation). High-standard infection control protocols should be applied to ensure the hiding box does not act as a fomite for possible infection transmission.

## How to apply this evidence in practice

The application of evidence into practice should take into account multiple factors, not limited to: individual clinical expertise, patient’s circumstances and owners’ values, country, location or clinic where you work, the individual case in front of you, the availability of therapies and resources.

Knowledge Summaries are a resource to help reinforce or inform decision-making. They do not override the responsibility or judgement of the practitioner to do what is best for the animal in their care.

## Clinical scenario

You are a veterinary nurse tasked with monitoring and feeding a ward of hospitalised cats. When you enter the ward, the wide-eyed cats hiss and are crouched tensely in their litter trays or burrowing under blankets. It is apparent that the cats are uncomfortable, fearful, and trying to hide so you avoid close contact, resulting in inadequate monitoring of some cats. Other veterinary nurses attempt to handle the cats but are injured as the fractious cats lash out. Due to this, some of the clinic's hospitalised cats cannot be handled, medicated, or undergo the planned diagnostic test or treatment in the timeframe planned. These delays and being under stress may compromise and negatively impact the cat's health. These experiences prompt you to consider other interventions to reduce the cats' stress. You consider whether a hiding box would make a significant difference to their stress levels so decide to review the best evidence on this topic.

## The evidence

The search identified five control trials relevant to the PICO question (Klintip et al., 2022; Arrandale & Buckley 2017a; Arrandale & Buckley 2017b; Wright & Baugh, 2018; Dewhurst & Reynolds, 2018). All five trials were stated by the authors as randomised control trials, however three used randomisation methods that were unclear, flawed, or aligned with non-randomised systematic allocation methods (Arrandale & Buckley 2017a; Arrandale & Buckley 2017b; Dewhurst & Reynolds, 2018). Four of the five studies suggested that the use of hiding boxes was associated with reduced stress for cats as evidenced by reduced cat stress scores (CSS) (Klintip et al., 2022; Arrandale & Buckley 2017a; Arrandale & Buckley 2017b; Wright & Baugh, 2018). No other measurements of stress such as salivary cortisol, temperature, or respiration rate, showed significant changes across the studies – except for Arrandale & Buckley (2017b), which found a statistically significant reduction in heart rate, but this could not be replicated in any other study. One study was inconclusive due to major limitations relating to small sample sizes and a flawed methodology (Dewhurst & Reynolds, 2018). Overall, the strength of the evidence was considered moderate as there were varying limitations across all studies (e.g. small sample sizes, short timeframes, unclear randomisation in three studies). However, all studies except Dewhurst & Reynolds (2018) found that hiding boxes were associated with reduced fear and stress.

## Summary of the evidence

**Arrandale & Buckley (2017a):** Towels versus hides: Which are best at reducing acute stress in the newly hospitalised domestic cat ( Felis sylvestris catus )? **Aim: **To identify whether towels over a cage or a box provided within a cage were better at reducing stress in the newly hospitalised cat.

Population:	Clinically healthy female and male domestic shorthair cats undergoing routine neutering surgery at a Southampton charitable veterinary hospital (UK). The median age of the cats was 1 year (ages ranged from 6 months to 6 years).
Sample size:	45 cats (male n = 27, female n = 18).
Intervention details:	The study was conducted over a 20-minute timeframe, prior to the administration of premedications and neutering surgery. Cats were housed in cattery cages stacked in two tiers, and were allocated by order of admission to one of the following treatment groups: Control Group (n = 15): No intervention provided. Hide Group (n = 15): The cage contained a cardboard box (40 cm x 25 cm x 25 cm). Towel Group (n = 15): The cage door was covered in a blanket with a 10 cm x 5 cm viewing hole. At first it seemed the cats were randomly allocated to each group as the researchers stated they allocated cats to the Control Group, Hide Group, or Towel Group randomly*, *“by the order of admission”.
Study design:	Randomised control trial.
Outcome Studied:	Behavioural and physiological indicators of the cats’ stress were studied: Each cat’s body temperature was recorded on admission and once more at 20 minutes. Each cat’s respiration rate was recorded on admission and once more at 20 minutes. Each cat’s heart rate was recorded on admission and once more at 20 minutes. A baseline Kessler & Turner Cat Stress Score (CSS) was recorded on admission and then measured every single minute up to 20 minutes.
Main Findings (relevant to PICO question):	For all groups median CSS significantly decreased over the 20 minutes of the study (Control Group: P = 0.002; Hide Group: P < 0.001; Towel Group: P < 0.001). There were no statistically significant findings in any of the groups end time respiration rate, heart rate or temperature. However, both Hide and Towel groups decreased in respiration rate and heart rate, whereas the Control Group did not decrease in respiration rate or heart rate. The median CSS of the Hide Group was significantly higher than the Control Group CSS at both 0 minutes (P < 0.001) and 20 minutes (P < 0.034). The median CSS of the Hide Group decreased more significantly than the Control cats (P = 0.003). This shows that the Hide Group incidentally had a higher median CSS than other groups at the start of the study and reduced the most of all groups. By the end the Hide Group’s CSS was still higher than other groups due to the Hide Group’s higher baseline CSS.
Limitations:	The study used small sample sizes. 15 cats in each group is a relatively small sample size to draw significant conclusions. Individual differences, such as personality, previous experiences with cage environments, or individual behaviours were more likely to influence results. Initially, the study stated the cats were randomly allocated into each group. However, the researchers also mention that each cat was allocated into the Control Group, then Hide Group, then Towel Group randomly* “*by the order of admission*”*. This is not true randomisation, because the first and last cats admitted were always going to be in the Control Group, and the second cat admitted would not be in the same group as the first cat admitted, and so on, which may have introduced other result-impacting factors. It is also not stated whether the allocation of intervention or cats into cage locations was randomised. This may have introduced potential bias into choosing which locations housed which intervention or cat. The study had a very small timeframe of observation. The cats were only observed for 20 minutes, which may not have been long enough to fully capture the effects of all interventions on stress levels. Some confounding factors, such as noise and activity in the ward, were not fully controlled. It is unclear whether the assessor of the stress parameters was blinded to the study. It is unclear whether the assessor of CSS was trained and practiced in the Kessler and Turner CSS system. The observer was present in the room while cat stress scoring the cats, rather than observing the cat through a window or by video recording. There were some complications in carrying out statistical analysis caused by the highly skewed nature of some of the data, variability between the cats parameters at 0 minutes, and the higher CSS of the Hide Group cats on admission. The study used a between-group design rather than within-subject design.

**Arrandale & Buckley (2017b):** The use of hides to reduce acute stress in the newly hospitalised domestic cat ( Felis sylvestris catus ) **Aim: **To identify if newly hospitalised cats would both use a hide, and show a reduction in stress levels when one was provided.

Population:	Clinically healthy female and male cats admitted for routine neutering surgery at a mixed animal private veterinary practice in Shropshire (UK). The cat's ages ranged between 5 and 7 months.
Sample size:	30 cats (male n = 16, female n = 14).
Intervention details:	Cats were allocated to one of the two treatment groups by systematic allocation in order of admission: Control Group (n = 15): No intervention provided. Hide Group (n = 15): Each cat’s cage contained a 40 cm x 25 cm x 25 cm cardboard box in one corner. The study was conducted over a 20-minute timeframe, prior to the administration of premedications and neutering surgery.
Study design:	Randomised control trial.
Outcome Studied:	The behavioural and physiological indicators of the cats’ stress were studied, as well the use of the hiding box: Each kennel was divided into four quadrants (A, B, C, and D). Each cat’s position in one of these quadrants was recorded every 30 seconds until 20 minutes. For the Hide Group only, the cat’s interaction with the box—either sitting on top of the box or inside it—was recorded every 30 seconds for up to 20 minutes. Each cat’s body temperature was taken on admission , and at 20 minutes. Each cat’s heart rate was recorded on admission and at 20 minutes. Each cat’s respiration rate was recorded on admission and at 20 minutes. Each cat’s Cat Stress Score (CSS) was recorded every 2 minutes for entirety of the 20-minute study.
Main Findings (relevant to PICO question):	Cats provided with a hiding box were significantly more likely to spend time in quadrant A (the quadrant containing the hiding box) than any of the other three quadrants (P < 0.001). Cats in the Control Group did not show a preference for any of the quadrants. Although, this was not statistically significant (P = 0.893). The cats who used the hiding box were more likely to go inside it, rather than on top of it (P < 0.001) with an average of 9 minutes out of 20 minutes in the box. At 0 minutes, the heart rate of the Hide cats was significantly higher than the Control cats (P = 0.030) The Hide Group cats had a significantly lower heart rate (P = 0.019) and CSS (P = 0.002) after 20 minutes than the Control Group, depicting a reduction in stress for the cats with hides. There were no statistical differences between the temperatures or respiration rates of the Hide and Control Groups.
Limitations:	The allocation of cats into treatment groups was not truly randomised. Instead, systematic allocation was used so the first cat to be admitted was allocated to the Control Group, the second to Hide Group, the third to Control Group, and so on. This is not true randomisation, which may introduce other factors or biases. This study only measured outcomes for up to 20 minutes, a small timeframe to identify true stress changes. The researchers state that this resulted in clear experimental limitations. A small sample size was used (15 cats in each group), meaning that individual differences, such as personality or previous experiences in the clinic, were more likely to influence results. It was not stated if the assessor was blinded to the purpose of the study or the treatment groups. Only two measurements of temperature, heart rate, and respiration rates (TPR) were taken. Specifically, the first measurement was taken at 0 minutes, and the second at 20 minutes. This may have meant the study missed recording any fluctuations in these outcomes between those times. Also, both of these TPR measurements were taken when the cat was outside of its kennel environment which may not accurately reflect the cat's stress levels when inside a cage with a hiding box. It was not stated whether the assessor of CSS was trained and practiced in the Kessler and Turner CSS system which would have improved the validity of the results. The observer was present in the room while scoring the cats. This may have meant the cat’s hide-seeking behaviour or stress was caused by the assessor being present in the room, rather than the interventions. It was not stated whether the allocation of interventions or cats into cage locations was randomised. This may have allowed for potential bias in choosing which locations had the interventions or cats. The study used a between-group design rather than within-subject design.

**Dewhurst & Reynolds (2018):** Does the Feline Fort ^®^ reduce stress in feline inpatients within a veterinary surgery and is it any better than a cardboard box or no hideaway at all? **Aim: **To compare the use of the Feline Fort to a disposable cardboard box, and also to assess whether having any hideaway at all reduced stress compared to having no hideaway at all within veterinary practice.

Population:	Clinically healthy female and male cats admitted for routine neutering surgery at a veterinary practice in Yorkshire (UK). The cats' ages ranged between 3 months and 18 years, with a median of 6 months and the mean age of 2.5 years.
Sample size:	21 cats (male n = 13, female n = 8).
Intervention details:	The study consisted of three treatment groups: Control Group (n = 8; male n = 5 female n = 3): No intervention provided other than usual environment outlined below. These cats were housed in a standard size kennel width 55 cm x depth 70 cm x height 55 cm. Cardboard Box Group (n = 7; male n = 5 female n = 2): A cardboard box measuring 39 cm x 41 cm x 30 cm (the same size as the Feline Fort^®^) was placed in each cage. Bedding was provided on top of and inside the box. The cats in this group were placed in a 60 cm x 88 cm x 64 cm sized kennel (larger than the Control Group). Feline Fort^®^ Group (n = 6; male n = 3 female n = 3): A plastic Feline Fort^®^ measuring 39 cm x 41 cm x 30 cm was placed inside the cage. Bedding was provided on top and inside the Feline Fort^®^. The cats in this group were placed in a 60 cm x 88 cm x 64 cm sized kennel (larger than the Control Group). All cats in the study were housed in a cat ward separated from other species. All cages contained a vet bed, litter tray, and a water bowl. The treatment applied to each cage was randomised. The study was conducted over a 30-minute timeframe, prior to the administration of premedications and neutering surgery. The study took place over 2 months.
Study design:	Randomised control trial.
Outcome Studied:	An ethogram containing a simplified cat stress scoring system and list of possible locations, was used to record the datapoints: Each cats Kessler & Turner Cat Stress Score (CSS) was recorded after 30 minutes, allowing for an acclimatisation period. After 30 minutes, an observer recorded each cat’s location in the cage. The observer could record the location of each cat in one of the five locations. All observations were recorded by a single observer. This observer was trained in the CSS scoring system.
Main Findings (relevant to PICO question):	The median CSS for all groups was 2. The Control Group, Cardboard Box Group, Feline Fort^®^ Group median CSS were approximately 1.8, 1.05, and 1.35, respectively. Five (38%) of the cats in the both the Cardboard Box Group and Feline Fort^®^ Groups combined used their intervention. Three (50%) of the six cats provided with the Feline Fort^®^ used it. There was no statistically significant difference in CSS between cats provided with either of the two hideaways (cardboard box and Feline Fort^®^) and the control group regardless of age or gender.
Limitations:	Small sample sizes were used in this study. This small sample size may have allowed individual animal differences to influence results. The researchers state a type II error, where an expected significant result is not returned, may have occurred due to this. It is unclear whether the cardboard boxes or Feline Fort^®^ was placed in the same location for all cages. No pilot study was undertaken before this study which would have helped to refine the study’s methodology and identify any confounding factors. It is unclear whether the observer measuring the stress behaviours was blinded to the allocation of interventions or purpose of the study. An oversimplified CSS system was used to measure feline stress. The researchers adapted the original CSS 7-level scoring system to a simplified 3-level stress system Simplifying the CSS system may have meant each cat’s stress was not correctly identified. The researchers stated that this method of measuring behavioural stress meant some behaviours fell between two possibilities leaving doubt as to which level to allocate. This study recorded CSS and cat location in cage after 30 minutes. This method meant that before the 30-minute mark exactly, CSS and use of the intervention would not have been recorded or reported, decreasing the study’s validity. Subjective behavioural markers of stress were examined rather than physiological biomarkers, possibly introducing observer bias. The observer entered the cat ward 1 minute before the outcomes were measured. This may have meant the cat’s recorded stress was in response to the new observer being in the room, rather than its cage environment. The study was conducted over a short timeframe of 30 minutes. This may have meant the cumulative effects of stress may have been overlooked. Only one CSS was taken for each cat in the study meaning that very little data was collected, which may have skewed the findings. Also, each cat’s behavioural stress observations were recorded at only one point in time which may have limited the accuracy and reliability of the results. There was no baseline CSS taken for each cat before interventions were provided, which meant there was no way of judging whether CSS reduced stress for each individual cat. If baseline data was taken it would allow for a comparison of stress levels before and after the intervention, providing insight into its effectiveness. It was not clear how the researchers allocated the cats to the cages containing randomised treatments. The study merely says the cats were randomly recruited from a healthy population of cats admitted for routine surgery, but it does not state how the cats were randomly allocated to the treatment groups. The researchers also state that this is a limitation as an independent person should have picked a number assigned to which treatment group the cat was to be allocated into. There were also confounding factors due to the study taking place in a busy practice over 3 months. The cats in the Cardboard Box and Feline Fort^®^ Groups had larger kennels than the Control Group as the cardboard box and Feline Fort^®^ were too large to fit into the standard sized kennels. This meant all three groups did not have consistent environments which is a confounding factor. The age of the participants varied greatly This is not not a major limitation for this study as any and all ages are hospitalised in veterinary clinics. However, it does introduce a possible confounding variable and potential for age-related differences to affect results. The results section of the study provided a fairly surface-level analysis. The results lacked any written results of the usage of the Cardboard Box Group. Two veterinary practice clinics were used as the setting for the study. This may have affected cage position and external environment as these were different in both practices. This may have introduced confounding factors and variability that decreases the validity of the findings. The study used a between-group design rather than within-subject design.

**Klintip et al. (2022):** First study on stress evaluation and reduction in hospitalized cats after neutering surgery **Aim: **To evaluate stress in hospitalised cats after neutering surgery using cat stress score (CSS) and salivary cortisol levels, including the impact of providing a hiding box (B) and/or administering a pheromone product to reduce stress.

Population:	Domestic cats hospitalised for recovery from neutering surgery (median age of cats was 1.4 ± 0.67 years). All cats were clinically assessed as healthy before the neutering surgery. The study was conducted from May 2020 to December 2021 in the Faculty of Veterinary Technology, Kasetsart University (Thailand).
Sample size:	80 cats (male n = 44, female n = 36).
Intervention details:	After housing the cats in cages with no interventions for 24 hours, each cats’ demeanour was assessed using a demeanour scoring system (DSS) to classify the cats into two groups, DSS1 – friendly (n = 39) and DSS2 – aggressive (n = 41). The assessors were blinded to the knowledge of which cat would be allocated to which treatment group. This study consisted of four treatment groups: Control Group (DSS1 n = 10, DSS2 n = 11): no additional enrichment in the cage. No hiding box or pheromone diffuser was located in the cage or room. Hiding Box Group (DSS1 n = 10, DSS2 n = 10): A hiding box measuring 39 cm x 25 cm x 25 cm was placed at the back of the cage. Pheromone Treatment Group (DSS1 n = 10, DSS2 n = 10): Cat cages were placed in a 220 × 640 × 250 cm room containing a pheromone diffuser (synthetic analog of the feline facial pheromone fraction F3, Feliway ^®^) for 7 days. Hiding Box-Pheromone Combination Group (DSS1 n = 9 DSS2 n = 10): Cat cages contained a hiding box and were located in the room containing the pheromone diffuser (synthetic analog of the feline facial pheromone fraction F3, Feliway ^®^) for 7 days. For all groups, each cage contained a water and food bowl, litter box and soft bedding. Three sides of each cage were covered with blankets to prevent visible access with other caged cats. The trial timeframe lasted for 8 days. Day 1 was defined as 24 hours after admission. Neutering surgery was completed on Day 2.
Study design:	Randomised control trial.
Outcome Studied:	Behavioural and physiological indicators of the cats’ stress were studied: The location of each cat in its cage was recorded twice daily. At the end of each day, the researchers weighed the food bowls to determine the daily food intake of each cat. On Days 1, 3, and 8 each cats’ bodyweight was recorded. Three saliva samples were taken from each treatment group on Days 1, 3, and 7. The cats to be sampled were randomly selected and fasted for at least 2 hours before sample collection. Assessors measured each cat’s stress score twice daily every day using the Kessler and Turner Cat Stress Score (CSS) system.
Main Findings (relevant to PICO question):	Providing a hiding box in all DSS1 (friendly cats) and DSS2 (aggressive cats) groups reduced CSS**,** with DSS2 cats showing a faster stress reduction. In all DSS1 groups, CSS continuously decreased, with statistical significance on the 2nd, 3rd, and 2nd day (this most likely this means the 4^th^ day but this is unclear due to a possible type error) after admission in the Hiding Box (P = 0.034), Pheromone (P = 0.017), and Hiding Box-Pheromone Combination (P = 0.012) treatment groups, respectively. In DSS1 cats, over the trial days, the Control Group had a higher median CSS than all other groups. The median for the Control Group was 2.76, while the median scores for the Hiding Box Group, Pheromone Group, and Hiding Box-Pheromone combination Group, were 2.28, 2.27, and 2.28, respectively. It was statistically significant that all DSS1 treatment groups had significantly lower CSS than the DSS1 control group (P < 0.001). In DSS2 (aggressive cats), CSS in the control group significantly decreased on day 6 (P < 0.001), whereas the Hiding Box (P = 0.004), Pheromone (P = 0.005), and Hiding Box-Pheromone Combination (P < 0.001) Group scores significantly decreased on the day 2. This shows that the cats calmed down faster when interventions were provided. When comparing positions inside the cage between control and treatment groups in both DSS1 and DSS2, there were was no significant differences (P > 0.05). Results showed no significant differences between salivary cortisol, food intake, bodyweight, or hide-seeking behaviour between treatment and control groups, so researchers determined these were ineffective for assessing stress in cats. Environmental enrichment was more effective in DSS2 cats than in DSS1 cats.
Limitations:	The study was unclear how they acquired participants. It only mentioned that owners informed researchers of their cats health before the study, indicating the cats to be used in the study may have been selected before admission. It is unclear where the Control and Hiding Box Groups were housed in the environment. The study merely states that the ‘wards’ were physically separated from the dog area with a stainless-steel cage (90 cm width × 75 cm height × 90 cm length). However, it is unclear what groups were located in the ‘wards’. Each treatment group had approximately 20 cats which is a relatively small sample size to draw significant conclusions. It is not stated how the researchers allocated the interventions or cats to their cage locations. If this was not randomised, it may have introduced bias. No information about the material of the box (whether plastic or cardboard) were provided. Observation times were sometimes infrequent so subtle changes or important datapoints may have been overlooked. For example, CSS was measured only once on days 2–8. Also, cortisol was only measured on day 1, 3, and 7. It was unclear at what time of day hide-seeking behaviour was observed. It is unclear at what specific times the researchers weighed the bowls for measuring food intake. The study states, “all cats … were kept in a dedicated room”, but also mentions the cats were physically separated from a dog area by a large steel cage. This would have prevented sightlines, but not the scent/sound of the dogs, potentially introducing a confounding factor. Dogs were not among the study participants but were present in a different location in the university facility. Only three cats from each group were randomly chosen for cortisol sampling. The timing of cortisol sampling may have reduced the validity of the findings. The researchers state that as cortisol has a half-life of 70–120 minutes, it is possible the inadequate timing of the sampling may have resulted in no statistical significance in cortisol levels. The study used a between-group design methodology where different groups of cats were used for each intervention. Instead, if appropriate, a within-subject design could have been utilised where each cat would have been exposed to every other intervention (with a long wash-out period in between) so each would serve as its own control. Additional stress measurements could have been used to determine the cats’ stress and efficacy of interventions. The researchers stated that salivary cortisol, food intake, bodyweight, or hide-seeking did not result in any statistical significance. The observer was present in the room while CSS scoring the cats, rather than observing the cat through a window or by video recording. This may have meant the cat’s hide-seeking behaviour or stress was caused by the assessor entering the room, rather than the interventions. The study divided cats into friendly and aggressive groups based off a demeanour questionnaire, but individual variability within these groups is still possible.

**Wright & Baugh (2018):** Effectiveness of providing a box, or partially covering the cage front, on reducing cat stress **Aim: **To investigate whether providing hospitalised cats with either a box or a partial towel cover to the front of the cage reduced stress levels, and whether each of these methods was sufficient in providing hiding opportunity.

Population:	Clinically healthy male and female pet cats admitted for routine neutering at a small animal veterinary practice in Shropshire (UK). The cats were aged between 5 months and 4 years, with a median age of 5 months.
Sample size:	42 cats (male n = 21, female n = 21).
Intervention details:	The study consisted of three treatment groups: Control Group (n = 14): No intervention provided. Box Group (n = 14): Plastic hiding box placed at back of the cage. The size of this box was not described in this study. Towel Group (n = 14): A partial towel cover was provided to cover one side of the cage door. The cats were allocated to each group randomly as the researchers randomly selected both a cage number and treatment number during admission to indicate where the cat would be housed and with what intervention. All cats in the study were subject to the normal cage set-up of a newspaper lined cage with two folded blankets at the back of the cage. All cats were housed in a cat-only ward with cages that did not face each other. The cats were housed for 60 minutes prior to the administration of premedications and neutering surgery. The entire study took place over 3 months.
Study design:	Randomised control trial.
Outcome Studied:	The behaviours of the cats were observed as indicators of stress. The usage of the hiding boxes was also examined: Each cat’s location (front, middle or back) was recorded every 15 minutes of the study until the 60-minute mark. Each cat’s Kessler & Turner Cat Stress Score (CSS) was recorded every 15 minutes of the study until the 60-minute mark. For Hide Group and Towel Groups only, it was recorded whether the cats were ‘using’ their respective treatments – either hiding inside the box or behind the towel. Each cat’s use of treatment was recorded every 15 minutes of the study until the 60-minute mark. The presence of hide-seeking behaviours (frequently repositioning themselves into corners or burying themselves under blankets/newspaper) was recorded using the 1/0 sampling method over 15-minute time intervals. 1/0 sampling is a non-quantitative method that merely shows that at one point inside a 15-minute time period (but for an unknown frequency or duration) the cat displayed a hiding behaviour. Please note hiding inside the box was not defined as a ‘hide-seeking behaviour’. All observations were recorded by a single observer who was a member of the nursing team. This observer was trained in the CSS scoring system.
Main Findings (relevant to PICO question):	Significant difference (P = 0.007) in median CSS between Control Group cats (n = 4.25) and Box Group cats (n = 2.5), with CSS of Hide Group cats being much lower. Significant difference (P = 0.004) in time spent exhibiting hide-seeking behaviours (hiding inside the box was not included as a ‘hide-seeking behaviour’) between Control Group (34%) and Box Group cats (2%). Box Group cats hid in the box 68% of the time spent in the hide box cage, indicating the hiding box would be used if offered. The Control Group cats had consistently highest median CSS across all four checks A statistically significant (P < 0.001) link was found between the presence of hide-seeking behaviours and elevated CSS. This link was strongest in the Control Group.
Limitations:	The sample size selected was 42 cats (14 in each group) which is a small size. All measurements were subjective behavioural observations rather than physiological parameters of stress. Using hide-seeking behaviours as indicators of stress alone is not a reliable way of assessing physiological and stress. Also, it is stated that it is possible some cats had high CSS but did not show hide-seeking behaviours due to behavioural inhibition. The CSS, use of treatment, and location in cage observations had a short data collection period, which may not capture the full extent or cumulative effects of stress. It is unclear whether the observer measuring the stress behaviours was blinded to the allocation of interventions or purpose of the study. The researchers definition of ‘hide-seeking behaviours’ made this measurement difficult to clearly identify or quantify, allowing for observer bias. Hide-seeking behaviour measurements were measured using the 1/0 sampling technique (over 15-minute time intervals). 1/0 sampling is a non-quantitative method that merely shows that at one point for an unknown frequency or duration, the cat displayed a hiding behaviour. This allows for observer errors or bias. The study used a between-group design rather than within-subject design.

## Appraisal, application and reflection

This Knowledge Summary aimed to identify whether the use of a hiding box as compared to no intervention is associated with reduced fear, stress, or anxiety for hospitalised cats. Hence, studies conducted in animal shelters were excluded during the search as these findings may have been influenced by other factors and are less relevant to clinical practice. Any studies conducted prior to 1 Jan 2015 were excluded to ensure only the most recent research relevant to today's veterinary practice were included.

Five studies relating to the PICO question were obtained (Klintip et al., 2022; Arrandale & Buckley, 2017a; Arrandale & Buckley, 2017b; Dewhurst & Reynolds, 2018; Wright & Baugh, 2018). All studies were primary research control trials. All five studies were defined by researchers as randomised. However, three of these studies had flawed group allocation methods and it was unclear if this was truly random or systematic allocation methods were used (Arrandale & Buckley., 2017a; Arrandale & Buckley., 2017b; Dewhurst & Reynolds, 2018). The researchers stated they allocated cats to the control group, hide group and/or other group “randomly, by the order of admission” (Arrandale & Buckley, 2017a; Dewhurst & Reynolds, 2018). However, this is reminiscent of systematic allocation, rather than true randomisation, which may have introduced confounding factors and bias.

Dewhurst & Reynolds (2018) found no link between reduced stress and the use of hides. However, these findings were inconclusive due to major limitations such as small sample sizes, oversimplified stress measurement tools, no baseline measurements, confounding factors (e.g. cage size of hideaway groups larger than Control Group), unclear randomisation methods, and a short study timeframe.

Klintip et al. (2022) had a more robust study design, clear randomisation, and utilised a wide range of objective and physiological stress markers. Klintip et al. (2022) also demeanour-scored the cats to determine the efficacy of hiding boxes in ‘aggressive’ cats versus ‘friendly’ cats. This study concluded that hiding boxes have a stress-reducing effect on both friendly and aggressive cats (Klintip et al., 2022).

The populations used in all five studies were cats undergoing neutering surgery. It is important to note that these studies did not research the use of hides in cats hospitalised for other health conditions, non-elective procedures, or in cats with behavioural disorders. Two studies used treatment groups with similar numbers of females and males (Arrandale & Buckley, 2017b; Wright & Baugh, 2018). However, two studies had more males than females (Arrandale & Buckley, 2017a; Dewhurst & Reynolds, 2018) and one study had more females than males (Klintip et al., 2022). This may have introduced a minor confounding factor in these studies as male and female cats can exhibit different stress responses or behavioural patterns (Tateo et al., 2021). Across the five studies, the median age of participants was between 6 months and 1 year old. All studies, except for Klintip et al. (2022), measured the cats’ stress during the preoperative phase, when the cats were awaiting their surgery (before premedications were given). Klintip et al. (2022) was the study that differed as the cats’ stress was measured for up to seven days after surgery.

All five studies had several limitations. Small sample sizes, unclear group randomisation methods, confounding factors, and short timeframes of measurement (most studies ranged from 20–60 minutes) were a recurring issue. These short timeframes may have caused the cumulative effects of stress to be overlooked, and potentially missed the full impact of the treatments. Four of the five studies had small sample sizes, ranging from 21–45 cats (Arrandale & Buckley, 2017a; Arrandale & Buckley, 2017b; Wright & Baugh, 2018; Dewhurst & Reynolds, 2018). Klintip et al (2022) had the largest sample size with 80 cats. However, the studies all had relatively small sample sizes compared to a recent study on hiding boxes in shelter cats, which involved 179 cats (Wojtaś et al, 2024). Hence, the reviewed studies may have faced challenges with statistical analysis, increased risk of Type II errors (potentially seen in Dewhurst & Reynolds (2018)), and increased variability due to individual differences among cats (such as personality, prior clinic experience, and personal preferences) (Mesquita et al., 2012; Cartlidge, 2020). However, conducting studies with larger sample sizes in veterinary clinics can be challenging due to constraints such as limited space, resources, and time.

All five studies also used a between-group design methodology rather than a within-subject design. If used, the within-subject design could have reduced variability due to individual cat characteristics, making it easier to attribute stress reductions directly to the interventions (Sedgwick, 2014; Wright & Baugh, 2018). However, it is not always possible to conduct a within-subject crossover study in veterinary research due to ethical and welfare considerations (Adami et al., 2023). Some studies also had minor confounding factors (Arrandale & Buckley, 2017a) had changing noise levels in the study environment), issues with stress measurements (Klintip et al., 2022) did not take cortisol samples every day), and lack of pilot studies conducted prior (Arrandale & Buckley, 2017a; Arrandale & Buckley, 2017b; Dewhurst & Reynold, 2018; Buckley & Mansbridge, 2017). In four of the five studies it was also not stated whether the allocation of interventions or cats into cage locations was randomised (Klintip et al., 2022; Arrandale & Buckley, 2017a; Arrandale & Buckley, 2017b; Dewhurst & Reynolds, 2018). Only one study clearly described that both the allocation of cats to treatment groups and the allocation of interventions to cage locations were randomised (Wright & Baugh, 2018). In studies where all interventions (control, hides, and others) were housed in the same room it would have been prudent to randomise interventions to cage locations to reduce bias and confounding factors.

Overall, however, these limitations are mitigated by the fact that four of the five studies (Klintip et al., 2022; Arrandale & Buckley, 2017a; Arrandale & Buckley, 2017b; Wright & Baugh, 2018) showed that hides are associated with reduced feline stress. This consistency across four different studies strengthens the overall conclusions.

Though each study had limitations, there were strengths in each study too. All studies had control groups, the interventions were well-implemented, and care was taken to minimise confounding factors as much as possible in the busy clinical setting. The study designs were undertaken by veterinary staff in an ethical and considerate manner using non-invasive or low-invasive measurement techniques.

The type and material of the hiding boxes used in the five studies varied. Three studies used cardboard boxes (Arrandale & Buckley, 2017a; Arrandale & Buckley, 2017b; Dewhurst & Reynolds, 2018), one used a plastic box (Wright & Baugh, 2018), another used the plastic Feline Fort^®^ (Dewhurst & Reynolds, 2018), and one study did not describe the material of the box (Klintip et al., 2022). While the focus of this paper is largely on determining the behavioural implications of using a hiding box for cats hospitalised in a veterinary clinic, the biosecurity risks and considerations for hygiene must also be considered. Dewhurst & Reynolds (2018) was the only study to mention cleaning procedures for their hiding boxes, noting that the plastic Feline Fort^®^ was cleaned before each use and the cardboard box was disposed and replaced for the next participant.

The studies varied greatly in their chosen feline stress measurements. One study utilised salivary cortisol, bodyweight, and food intake as stress markers (Klintip et al., 2022). Another measured the presence of hide-seeking behaviours (frequent repositioning into corners or burrowing under blankets) (Wright & Baugh, 2018). Two studies measured the cats’ heart rates, respiration rates, and body temperatures (Arrandale & Buckley, 2017a; Arrandale & Buckley, 2017b). However, all five studies utilised a cat stress score (CSS) system to measure behavioural signs of stress. Interestingly, in all studies where they were measured, objective physiological stress measurements showed no statistically significant differences between control and hiding box groups. Only in one study out of the two that measured heart rate found a statistically significantly lower heart rate in the Hiding Box Group (Arrandale & Buckley, 2017b). However, Arrandale & Buckley (2017a) stated that this may be due to confounding factors. Additionally, four of the five studies recorded each cat’s usage and interaction with the hiding box (Klintip et al., 2022; Arrandale & Buckley, 2017b; Wright & Baugh, 2018; Dewhurst & Reynolds, 2018). Three studies concluded that cats were likely to use the box if it was provided and preferred to hide inside the box (Arrandale & Buckley, 2017a, Arrandale & Buckley, 2017b; Dewhurst & Reynolds, 2018).

All five studies utilised the Kessler & Turner (1997) CSS, except Dewhurst & Reynolds (2018) who simplified and modified it. The Kessler & Turner (1997) CSS is a non-invasive subjective behavioural assessment tool used to measure stress levels in cats.  This CSS system has been used reliably to measure feline stress in shelter environments (Hirsch et al, 2021; Vojtkovská et al., 2020; Van der Leij et al., 2019). However, research shows that cat stress scoring is currently underutilised in the veterinary clinic setting (Hill, 2023).

Interestingly, in the four studies that utilised the unmodified Kessler & Turner (1997) CSS, evidence of reduced stress in cats with hiding boxes was found (Arrandale & Buckley, 2017a; Arrandale & Buckley, 2017b; Klintip et al., 2022; Wright & Baugh, 2018). This again suggests that the Kessler & Turner (1997) CSS system is a reliable method to measure behavioural signs of stress in felines (Vojtkovská et al., 2020; Hirsch et al, 2021; Van der Leij et al., 2019). Notably, in the four studies using the unmodified Kessler & Turner CSS, behavioural stress signs reduced with the use of hides but measurable physiological stress changes could not be consistently identified. This may have been that studies’ limited sample sizes, confounding factors (e.g. removing cats from cages and handling during measurement) or the specific physiological stress measurements used in these studies were not ideal. Also, behavioural indicators may be more reliable and sensitive for short-term studies, whereas physiological changes may take longer to manifest and were likely missed in short study timeframes (Arrandale & Buckley, 2017b; Hirsch et al. 2021). Notably, this Knowledge Summary raises questions about the relationship between behaviour and physiology in cats under stress, as it was found that outward behaviours do not always correspond with measurable physiological changes. It is also important to note that the reliance on subjective observations in these studies means that while consistent evidence of stress reduction was found, there is potential for bias or interpretational differences.

Fear/stress in animals is defined as a physiological and behavioural adaptive response to threatening stimuli (Riemer et al., 2021; Lloyd, 2017). Physiologically, the hypothalamic-pituitary-adrenal axis is activated, releasing cortisol, and increasing heart rate, temperature, blood pressure and other bodily functions (Riemer et al., 2021). Behaviourally, the animal may display a ‘fight, flight, or freeze’ response to avoid or cope with the threat (Riemer et al., 2021). A cat’s stress response can range from mild signs of stress (e.g. mildly tense body) to extreme panic and terror (e.g. yowling) (Lloyd, 2017; Kessler & Turner, 1997).

The veterinary clinic environment can be highly stressful for cats. Firstly, they must travel to the clinic which can be a stressful event for owner and cat (Caney et al., 2022). Once the cat arrives in the clinic, they are faced with an unfamiliar environment and possible separation from their owner, which can trigger negative emotions (Taylor et al., 2022). These compounding stressors leads to a concept known as ‘stressor stacking’, where stressors accumulate during preparation for the veterinary visit at home, and continue during admission, physical examination, treatment, hospitalisation, and even once the cat has returned home (Taylor et al., 2022). This is a real challenge for caring for feline in-patients as described in the clinical scenario outlined above. The increasing physiological and behavioural stress of the patient can lead to misleading clinical findings, prolonged recoveries, difficult handling for staff (and possible injuries), treatment delays, and ultimately compromise feline health and wellbeing (Taylor et al., 2022; Lloyd, 2017; Riemer et al., 2021).

The evidence reviewed in this Knowledge Summary suggests that in domestic cats hospitalised in the veterinary clinic, the use of hiding boxes compared to no intervention is associated with reduced stress. Four of the five studies found that the use of a hiding box as compared to no intervention was associated with reduced stress (Klintip et al., 2022; Arrandale & Buckley, 2017a; Arrandale & Buckley, 2017b; Wright & Baugh, 2018).

It is well-acknowledged that cats have an instinct to hide when faced with stress-inducing stimuli.  Therefore, hiding boxes may help reduce stress for cats by giving them a sense of security, control, and comfort (Taylor et al., 2022). They may also limit the awareness of environmental triggers (e.g. disrupting sightlines of other cats). It is also thought that allowing an animal to display normal behaviours can help minimise negative emotions or promote positive ones (Taylor et al., 2022). This concept was supported by Wright & Baugh (2018) which found a link between cats in the Control Group exhibiting hide-seeking behaviours (e.g. burrowing under newspaper or positioning into cage corners) and having higher CSS. Wright & Baugh (2018) also observed that the Control Group cats spent an average 34% of the 60-minute timeframe exhibiting hide-seeking behaviours whereas the Hiding Box group cats spent less time (an average of 2% of the 60-minute timeframe) exhibiting hide-seeking behaviours.

Therefore, after reviewing the evidence, it is recommended that veterinary clinics consider providing each cat with a hiding box in addition to the normal feline cage environment (e.g. bedding, litter trays, food/water bowls.). Specifically, this can be done using a plastic box, plastic bucket, cat carrier, or single-use modified cardboard box (Taylor et al., 2022; Lloyd, 2017). It is imperative that the boxes are clean, large enough to fit comfortably within the cage, feline-friendly and that there are no sharp sides that may harm patients. It is important to note that in all five studies there was no hiding box groups where increased stress was observed (Arrandale & Buckley, 2017a; Arrandale & Buckley, 2017b; Klintip et al., 2022; Wright & Baugh, 2018 ;Dewhurst & Reynolds, 2018). However, when providing hiding boxes to feline patients it is always prudent to observe the patient and consider whether the intervention is having the desired effect (Wright & Baugh, 2018).

The material choice of the hiding box may impact the ease of cleaning and efficacy of disinfection. It is known porous materials (such as cardboard) are more difficult to clean and disinfect and may act as a fomite for disease or infection transmission if reused (Stull et al, 2018). Hence, hiding boxes made from cardboard should be disposed after use. Non-porous materials (such as some plastics) are easier to clean and disinfect so can be reused (Traverse & Aceto, 2022). Taylor et al. (2022) states that cat ward cages should contain non-permeable and easy to disinfect materials such as laminated or moulded plastic. It is imperative that high-standard infection control and hygiene protocols are undertaken whenever using hiding boxes, just as with other veterinary clinic environments, equipment, and surfaces.

In some cases, the use of a hiding box may be inappropriate for some cats. For example, some patients may require continual clear visualisation for monitoring. Additionally, animals with fears of hiding boxes or small spaces should not be provided with one. Therefore, it is recommended that a hiding box is added to a standardised cage set-up protocol, with practitioners deciding whether to use it or not as they assess each individual case. It is also important to note that the use of hiding boxes has not been well researched for use in unwell patients or patients with behavioural disorders, so their use in these specific situations is currently unclear. Alternatively, the hiding box could be reserved for more anxious or aggressive cats in the clinic. Klintip et al. (2022) proved that hiding boxes were more effective in cats with an ‘aggressive’ demeanor as these cats showed a faster stress reduction than the ‘friendly’ cats.

Three out of the five studies support the use of a hiding box to reduce stress in the preoperative phase (when the patient was awaiting surgery but had not been given premedications), so it is specifically recommended to be used at this time (Arrandale & Buckley, 2017a; Arrandale & Buckley, 2017b; Wright & Baugh, 2018). Only one study researched the use of a hiding box for a longer-term period of 8 days (Klintip et al., 2022). While the box proved useful in reducing CSS over that time, this shows there is currently limited evidence supporting their use in longer-term hospitalised patients (Klintip et al. 2022). It should also be mentioned that all studies focused on cats undergoing neutering surgery so naturally they had younger patients as their participants. Hence, the findings of these studies are more representative of this age group.

Applying this evidence has few barriers as hiding boxes are more affordable, easily stored and are non-invasive. However, there may be some challenges associated with making changes to usual clinic protocols, and it can be difficult to ensure the box fits comfortably inside cages. Dewhurst & Reynolds (2018) found that hiding boxes the same size as the Feline Fort^®^ (39 cm x 41 cm x 30 cm) could not fit inside their standard size kennels (55 cm x 70 cm x 55 cm) alongside food/water bowls, litter trays, and resting spaces.

Currently, studies on the use of hiding boxes in clinical settings are limited and have small sample sizes, so further studies are required to strengthen and refine their use. Future research could involve randomised control trials with larger sample sizes, clearer randomisation, minimised confounding factors, targeted sample populations (e.g. unwell cats, anxious cats), and longer timeframes. Also, future studies could utilise additional non-invasive measurements/biomarkers of feline stress, such as blood pressure, infrared thermography, faecal glucocorticoid metabolite, or other cortisol measurements (e.g. salivary, urinary, or hair cortisol), which have been utilised in similar studies focusing on shelter cats (McCobb et al., 2005; Ellis et al., 2021; Klintip et al., 2022; Wojtaś et al., 2024;). As discussed, Klintip et al. (2022) used cortisol to measure stress. However, the salivary cortisol samples were collected irregularly, only three cats were chosen for sampling from each group (containing 10 cats), and the timing of sample testing was inadequate (Klintip et al., 2022).

Notably, the use of hiding boxes has been recently recommended in the 2022 International Society of Feline Medicine and the American Association of Feline Practitioners Cat Friendly Veterinary Environment Guidelines consensus source which is used to inform evidence-based practice (Taylor et al., 2022). Interestingly, however, these guidelines only cited studies conducted in shelter cats when describing hiding box recommendations. Therefore, this Knowledge Summary intends to help clarify the efficacy and use of hiding boxes in veterinary practice.

## Methodology

**Search Strategy:** 

Databases searched and dates covered:	CAB Abstracts on CABI Digital Library Platform [Jan 2015–Feb 2025] PubMed on NCBI platform [Jan 2015–Feb 2025] Scopus on Elsevier platform [Jan 2015–Feb 2025]
Search strategy:	CAB Abstracts: ((hospitalised OR hospitalized) AND (cat OR cats OR feline OR felines OR "Felis catus")) AND ((hides OR boxes) OR ((hidey OR hiding OR cubby) AND (holes OR boxes OR spaces OR places))) AND (anxiety OR fear OR stress) PubMed: ((hospitalised OR hospitalized) AND (cat OR cats OR feline OR felines OR "Felis catus")) AND ((hides OR boxes) OR ((hidey OR hiding OR cubby) AND (holes OR boxes OR spaces OR places))) AND (anxiety OR fear OR stress) Scopus: ((hospitalised OR hospitalized) AND (cat OR cats OR feline OR felines OR "Felis catus")) AND ((hides OR boxes) OR ((hidey OR hiding OR cubby) AND (holes OR boxes OR spaces OR places))) AND (anxiety OR fear OR stress)
Dates searches performed:	11 February 2025

**Exclusion / Inclusion Criteria:** 

Exclusion:	Non-english.language publications Not peer-reviewed. Published before January 2015. Case study/reports/series, opinion pieces, cohort studies, case-control studies, cross-sectional studies, textbooks, books, secondary research (systematic reviews, meta-analysis). Studies not relating to the sample population Studies unrelated to the PICO question. Studies that did not measure fear or stress reduction but focused on different outcomes (e.g. appetite increase).
Inclusion:	Primary research studies (randomised and non-randomised control trials) containing at least one control group and one group with the hiding box intervention. Studies with sample population of domestic cats hospitalised in veterinary clinic, veterinary hospital, or veterinary research hospital. Studies using objective behavioural or physiological measurements for stress/fear.

**Search Outcome:** 

Database	Number of results	Excluded – not relevant to the PICO question	Total relevant papers
CAB Abstracts	48	43	5
PubMed	2	1	1
Scopus	5	4	1
Total relevant papers when duplicates removed			5

## Acknowledgements

I would like to thank to Jennifer Hamlin for her constant encouragement and mentorship. Thank you also to the editors and peer reviewers of Veterinary Evidence who gave up their time to make this paper the best it could be. I would also like to thank my family, particularly my sister, who were fantastic sounding boards during the writing of this paper. Primarily though, I must heartfully acknowledge my two best friends, my precious beloved furbaby G, and my beautiful king J, who are the reason for everything.

## ORCiD

Leah Foster: https://orcid.org/0009-0005-5875-5558

## Conflict of Interest

The author declares no conflicts of interest.

## Use of artificial intelligence

The author declares that no generative artificial intelligence (AI) tools were used in the writing of this manuscript. All content, interpretations, and conclusions were produced entirely by the author without AI assistance.
